# Role of ATG4 Autophagy-Related Protein Family in the Lower Airways of Patients with Stable COPD

**DOI:** 10.3390/ijms25158182

**Published:** 2024-07-26

**Authors:** Francesco Nucera, Antonino Di Stefano, Fabio Luigi Massimo Ricciardolo, Isabella Gnemmi, Cristina Pizzimenti, Francesco Monaco, Giovanni Tuccari, Gaetano Caramori, Antonio Ieni

**Affiliations:** 1 Department of Biomedical, Dental, Morphological and Functional Imaging Sciences, Section of Pneumology, University of Messina, 98125 Messina, Italy; francesco.nucera@unime.it; 2 Istituti Clinici Scientifici Maugeri, IRCCS, Respiratory Rehabilitation Unit of Gattico-Veruno, Section of Pneumology, Laboratory of Cytoimmunopathology in Cardio Respiratory System, 28013 Gattico-Veruno, Italy; antonino.distefano@icsmaugeri.it (A.D.S.); isabella.gnemmi@icsmaugeri.it (I.G.); 3 Department of Clinical and Biological Sciences, Severe Asthma, Rare Lung Disease and Respiratory Pathophysiology Unit, San Luigi Gonzaga University Hospital, University of Turin, 10043 Orbassano, Italy; fabioluigimassimo.ricciardolo@unito.it; 4 Department of Human Pathology in Adult and Developmental Age ‘Gaetano Barresi’, Section of Pathology, University of Messina, 98125 Messina, Italy; cristinapizzimenti86@gmail.com (C.P.); giovanni.tuccari@unime.it (G.T.); 5 Department of Biomedical, Dental, Morphological and Functional Imaging Sciences, Section of Toracic Surgery, University of Messina, 98125 Messina, Italy; francesco.monaco@unime.it; 6 Department of Medicine and Surgery, Sections of Pneumology, University of Parma, 43126 Parma, Italy; gaetano.caramori@unipr.it

**Keywords:** autophagy, chronic obstructive pulmonary disease, ATG4, treatment, lung disease

## Abstract

Autophagy is a complex physiological pathway mediating homeostasis and survival of cells degrading damaged organelles and regulating their recycling. Physiologic autophagy can maintain normal lung function, decrease lung cellular senescence, and inhibit myofibroblast differentiation. It is well known that autophagy is activated in several chronic inflammatory diseases; however, its role in the pathogenesis of chronic obstructive pulmonary disease (COPD) and the expression of autophagy-related genes (ATGs) in lower airways of COPD patients is still controversial. The expression and localization of all ATG proteins that represented key components of the autophagic machinery modulating elongation, closure, and maturation of autophagosome membranes were retrospectively measured in peripheral lungs of patients with stable COPD (*n* = 10), control smokers with normal lung function (*n* = 10), and control nonsmoking subjects (*n* = 8) using immunohistochemical analysis. These results show an increased expression of ATG4 protein in alveolar septa and bronchiolar epithelium of stable COPD patients compared to smokers with normal lung function and non-smoker subjects. In particular, the genes in the ATG4 protein family (including ATG4A, ATG4B, ATG4C, and ATG4D) that have a key role in the modulation of the physiological autophagic machinery are the most important ATGs increased in the compartment of lower airways of stable COPD patients, suggesting that the alteration shown in COPD patients can be also correlated to impaired modulation of autophagic machinery modulating elongation, closure, and maturation of autophagosomes membranes. Statistical analysis was performed by the Kruskal–Wallis test and the Mann–Whitney U test for comparison between groups. A statistically significant increased expression of ATG4A (*p* = 0.0047), ATG4D (*p* = 0.018), and ATG5 (*p* = 0.019) was documented in the bronchiolar epithelium as well in alveolar lining for ATG4A (*p* = 0.0036), ATG4B (*p* = 0.0054), ATG4C (*p* = 0.0064), ATG4D (*p* = 0.0084), ATG5 (*p* = 0.0088), and ATG7 (*p* = 0.018) in patients with stable COPD compared to control groups. The ATG4 isoforms may be considered as additional potential targets for the development of new drugs in COPD.

## 1. Introduction

Autophagy is an intracellular pathway that can be activated by several stimuli (e.g., hypoxia, oxidants, DNA damage, intracellular protein aggregates, damaged organelles, or intracellular pathogens) essential for the homeostasis and survival of normal cells through lysosomal degradation of damaged organelles [1,2]. In addition, autophagy has an important role in the host’s defenses against infectious pathogens inducing the degradation of several bacteria, fungi, protozoa, and viruses [3,4].

Three major types of autophagy have been described [5] macroautophagy, microautophagy, and chaperon-mediated autophagy, which converge in the degradation of several intracellular components mediated by various hydrolases such as proteases, esterase, nucleases, and sulfatases depending on the composition and structure of the target [6]. In particular, macroautophagy represents the most studied autophagy pathway in human cells, characterized by the formation of a double membrane that can exclude the degrading component from the rest of the cytosol forming the autophagosomes that finally are fused with the secondary lysosomes [7]. This process is divided into a multistep process summarized in three main phases [3]: (a) induction and nucleation started with the nucleation of a crescent-shaped double membrane termed phagophore through activation of the selective autophagy receptors (SARs), such as p62 (also called sequestosome-1; SQSTM1) [8]; (b) elongation, cargo recruitment, and closure characterized by the nucleated phagophore membrane surrounding the cytoplasmic target expand, forming an autophagic vacuole (autophagosome) [3]. This phase of enlargement is mediated by two ubiquitin-like (Ubl) conjugation systems, such as the ATG5–ATG12 conjugation system, and in particular ATG8 [3]. In humans, this system is known as the protein complex microtubule-associated protein 1 light chain 3 (LC3), which includes two isoforms like LC3A and LC3B. LC3 is produced as a precursor termed “Pro-LC3” that is trunked by the proteolytic activity of the ATG4 protein family (which includes four isoforms as -A; -B; -C; -D) forming the LC3-I fragment, which can interact through ATG3 and ATG7-mediated activity with phosphatidylethanolamine (PE), producing the LC3-II complex though a “lipidation” process [9]. LC3-II is the active form of LC3 and is localized on both internal and external membranes mediating autophagosomes expansion and closure [3,4,9]. Lastly, the third phase is represented by: (c) maturation and fusion, in which the maturation of autophagosomes to degradative autolysosomes is mediated by the fusion with endolysosomal compartments [7]. In this phase, LC3-II is reconverted in LC3-I by the ATG4 proteins family through its delipidation activity, mediating LC3 detachment from the autophagosome membrane [9,10,11]. In this way, a recycle of LC3-I happens, allowing and mediating closed autophagosome fusion with lysosomes through the microtubule center organization (MTCO). Successively, MTCO moved closed and mature autophagosomes where fusion with the lysosomes occurs (forming autophagolysosome). At this point, the lysosomal enzymes may degrade the content, which is then released into the cytoplasm to be recycled in other cellular pathways [12]. 

Relationships between autophagy and pulmonary disease have been considered an interesting topic arising in some reviews, either considering acute and/or chronic obstructive lung conditions [12,13,14,15]. Focusing on the autophagy pathway in respiratory diseases, it has been documented that autophagy is stimulated in response to various stimuli of acute lung injury (ALI), in which a loss of ATG genes, such as Atg7, Atg5, and Atg4B, experimentally aggravates this condition [12]. Moreover, autophagy seems to be involved in the pathogenesis of asthma through adaptive immune responses, eosinophilic airway inflammation, and airway remodeling [16]. Furthermore, the deficiency of ATGs could promote the deposition of extracellular matrix in lung fibroblasts and increase the process of fibrosis in interstitial lung disease [16].

Currently, data regarding autophagy in COPD are still controversial, even though recent studies have shown that although cigarette smoke (CS) represents an important stimulus to induce autophagy activation. This signaling pathway is impaired in COPD as although the expression of LC3 is increased in COPD patients and smokers with normal lung function compared to non-smokers controls, COPD patients also show an enhanced expression of p62 that is well-known to be a marker correlated to impaired autophagy and increased intracellular accumulation of damaged organelles and noxious molecules that usually are destroyed through the autophagic flux [17], suggesting that other autophagic mediators can have a key role in the modulation and in the effectiveness of intracellular autophagy. The purpose of the present study is to morphologically analyze different components of the autophagosome by immunohistochemistry as well as the expression of several ATG proteins in biological lung samples obtained from patients affected by stable COPD compared with smokers with normal lung function and non-smoker subjects. 

## 2. Results

**Figure 1:**
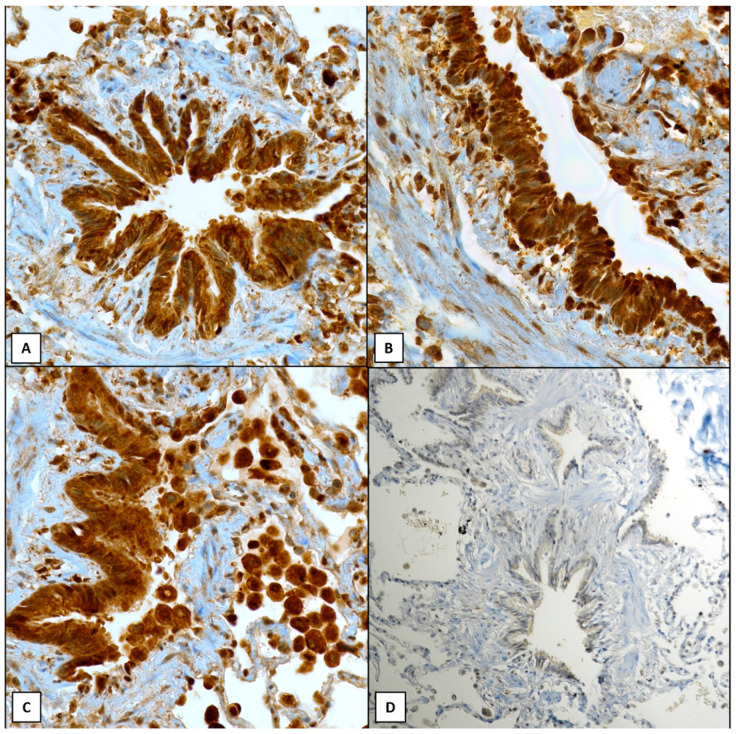
Immunoreactivity of ATGs in bronchial epithelium: a diffuse strong nuclear and cytoplasmic (score 6) staining was encountered with ATG4A (**A**, 400×), ATG4D (**B**, 400×), ATG5 (**C**, 400×) in patients with stable COPD; note the absence of immunoreaction in CNS/CS patients (**D**, 200×). Nuclear hemalum counterstain was made in all cases.

All significant positive data are summarized in Table 1. The immunoreactivity was analyzed in its distribution, either nuclear or cytoplasmic, mainly concerning the bronchial epithelium as well as the alveolar lining. In detail, a statistically significant increased expression of ATG4A (*p* = 0.0047) (Figure 1A), ATG4D (*p* = 0.018) (Figure 1B), and ATG5 (*p* = 0.019) (Figure 1C) was documented in the bronchiolar epithelium in patients with stable COPD compared to CS and CNS groups (Figure 1D). 

**Figure 2:**
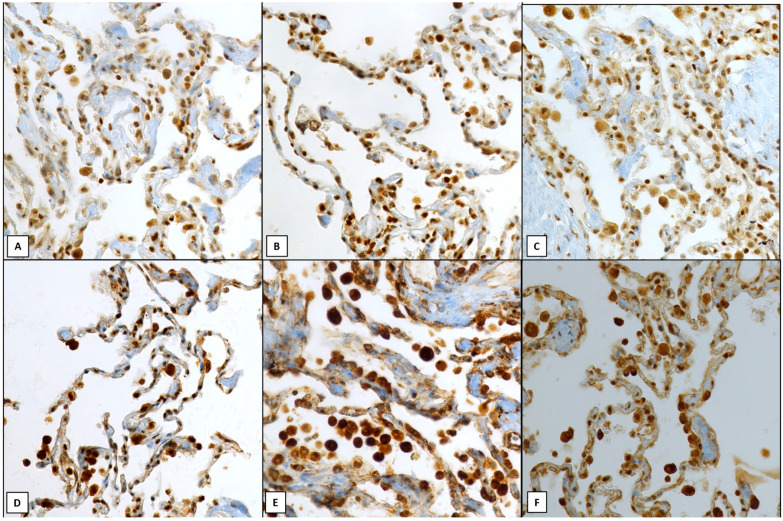
Immunoreactivity of ATGs in alveolar lining: a diffuse strong nuclear and cytoplasmic (score 5–6) staining was encountered with ATG4A (**A**, 400×), ATG4B (**B**, 400×), ATG4C (**C**, 400×) ATG4D (**D**, 400×), ATG5 (**E**, 400×) and ATG7 (**F**, 400×) in patients with stable COPD, Note also the immunopositivity in alveolar macrophages. Nuclear hemalum counterstain was made in all cases.

Similar statistically significant values were encountered in the immunoexpression of ATG4A (*p* = 0.0036, Figure 2A), ATG4B (*p* = 0.0054, Figure 2B), ATG4C (*p* = 0.0064, Figure 2C), ATG4D (*p* = 0.0084, Figure 2D), ATG5 (*p* = 0.0088, Figure 2E), and ATG7 (*p* = 0.018, Figure 2F) in alveolar lining of stable COPD compared to CS and CNS patients.

By contrast, no significant differences in other compartments of lower airways including lung smooth muscle cells, alveolar capillary, endothelial cell veins, and alveolar macrophages were found in terms of immunoexpression of ATG4A, ATG4B, ATG4C, ATG4D, ATG5, and ATG7. Moreover, any further significant differences in the expression of ATG2A, ATG2B, ATG3, ATG10, ATG12, ATG14 (Thr429), ATG14, and ATG16L were found in all other cellular compartments of COPD patients compared to CNS and CS.

## 3. Discussion

The role of autophagy in chronic inflammatory diseases including COPD is still now controversial [18], although it is well known that autophagic flux may change during chronic inflammation, with contrasting data concerning either an increase or decrease of the autophagic cascade in COPD [12,18,19]. However, growing evidence suggest that autophagy is induced by cigarette smoke, but it tends to be impaired during COPD showing that in stable COPD patients impaired macroautophagy can play a key role in maintaining the pro-inflammatory state of the lungs, in damaging and inducing apoptosis in lung cells, leading to pulmonary emphysema and worsening of the disease [20]. Furthermore, impaired autophagy in alveolar macrophages is correlated with their altered phagocytosis and killing capacity and consequently with decreased defense against several pathogens, such as bacteria and viruses [21]. In particular, autophagy in pulmonary myeloid cells, including alveolar macrophages, is known to prevent excessive immune responses and inflammation under pathological conditions, such as endotoxemia, cystic fibrosis, and hemorrhagic shock [22]. On the other hand, the role of ATGs in tissue-resident cells, such as muscle cells, endothelial cells, and fibroblasts should be not emphasized since no significant differences have been observed in these compartments in relation to all classes of ATGs. Therefore, it may be suggested that autophagy may determine any increased susceptibility to infections and consequently any increased risk of COPD exacerbation, worsening the quality of life and prognosis of COPD patients. 

Recently, these data have been confirmed by analyzing through several molecular methods the lower airways of stable COPD patients with different grades of severity, showing increased expression of LC3A + LC3B in both smokers with normal lung function and in COPD patients compared to non-smoker subjects, suggesting thus an enhanced autophagic flux [17]. Moreover, an increase of p62 was found in COPD patients compared to controls, suggesting that the autophagic flux is also ineffective in COPD [17].

The investigation has extensively analyzed the immunoexpression of a larger panel of ATGs in order to verify if a further increased expression of them may be present in COPD patients. Although some biases are present in this study, such as the size of the analyzed cohort as well as the use of an exclusive immunohistochemical approach, and the absence of molecular/genetic data of patients, data are in accordance with several studies that have previously demonstrated this kind of alteration in autophagic flux in other chronic inflammatory lung diseases, including also lung cancer that is strictly correlated to COPD representing an independent risk factor for lung cancer development [9,23,24]. 

Interestingly, we extended previously reported data [17], showing an increased expression of selected AGTs such as AGT5, ATG7, and in particular, the ATG4 protein family that has a key role in the process of elongation, cargo recruitment, and closure of phagophore through both the transformation of the pro-LC3 in active LC3, but also in the delipidation and recycling of LC3 [18,24]. These data are consistent with the previous ones appeared in literature and they could explain both the increased expression of LC3 in COPD as well as the ineffective autophagic flux characterizing COPD since the increased expression of these ATGs (such as ATG5, ATG7, and ATG4 protein family) involved in activation and recycling of LC3 can occur in an accelerated, uncoordinated and impaired autophagic flux. Secondly, we also demonstrated that the most involved ATGs in COPD are represented by several ATG4 isoforms. It is well known that during physiological conditions, the most important ATG4 is represented by ATG4B, whereas the other isoforms are poorly expressed, and not effective compared to ATG4B. However, it is known that the other isoforms can replace the ATG4B absence of inactivation [18,25] and this could enforce the present results, suggesting that the impaired autophagic flux can be mediated by the increased several ATG4 isoforms, which are less effective compared to the ATG4B isoform. Thirdly, it can be hypothesized that the impaired expression of the ATG4 isoforms could be related to oxidative stress. In fact, is noteworthy that oxidative stress is increased in COPD and has a key role in the development and evolution of this disease. Moreover, the ATGs, and particularly the ATG4 isoforms, can be inactivated by oxidative stress [9,24]. This furtherly enhances the hypothesis that the increased expression of several ATG4 isoforms could be a compensatory mechanism during COPD, which, however, results in ineffective autophagy.

## 4. Materials and Methods

### 4.1. Subjects

The subjects involved in this study were recruited from the Respiratory Medicine Unit of the Istituti Clinici Scientifici Maugeri (Veruno, Italy) and the Department of Clinical and Biological Sciences of the San Luigi Gonzaga University Hospital (Orbassano, Italy). The study has been approved by the institutional review boards of Istituti Clinici Scientifici Maugeri (protocol p112), by the ethical committee of the San Luigi Gonzaga University Hospital (protocol n. 9544/2019) and by the ethical committee of Policlinico “G.Martino” of Messina, Italy (non-profit experimental study 55-17). All patients have signed an informed consent. Ten were smokers with normal lung function, eight were non-smoker subjects, and ten subjects were stable COPD patients (Table 2). All former smokers had stopped smoking for more than one year. COPD patients and the control group were comparable in age and sex. Ex-smokers must have stopped smoking for at least a year. None of the subjects received glucocorticoids (systemic or inhaled), theophylline, antibiotics, or antioxidants in the month before surgery. All subjects had not undergone pre-operative chemotherapy and/or radiotherapy and had not received other immunosuppressive drugs. Within 30 days prior to surgery, each patient had an accurate medical history (including smoking habits, long-term home drug therapy taken, and the presence or absence of chronic bronchitis) and was subjected to spirometry with an acute bronchodilation test and routine chest computed tomography (usually with contrast). COPD diagnosis was posed as an exclusion diagnosis in subjects > 40 years with a smoking history > 20 p-y and persistent airflow obstruction (FEV1/FVC ratio < 0.7) after the exclusion of the many other known causes of persistent airflow limitation such as bronchial asthma with persistent airflow limitation, bronchiectasis, previous TB, ILDs, occupational exposures (coal mine dust, silica, welding fume, textile dust, agricultural dust, cadmium fume), cryptogenic organizing pneumonia, pulmonary Langherans’ cell histiocytosis, and lymphangioleiomyomatosis.

All subjects did not undergo preoperative chemotherapy and/or radiotherapy and had not been treated with bronchodilators, theophylline, antibiotics, antioxidants, and/or glucocorticoids in the month prior to surgery.

### 4.2. Lung Function Tests and Volumes

Pulmonary function tests were performed as previously described [25,26] according to published guidelines [27]. Pulmonary function tests included measurements of FEV1 and FEV1/FVC under baseline conditions in all the subjects examined (6200 Autobox Pulmonary Function Laboratory; Sensormedics Corp., Yorba Linda, CA, USA). Pulmonary function tests were performed during the pre-operative surgical visit for the removal of the pulmonary nodule. All subjects involved in the study performed pulmonary function tests and patients performed long-term inhaled therapy they suspended inhalation therapy for at least one day before performing the pulmonary function tests. In order to exclude the reversibility of airflow obstruction (suggesting asthma) and post-bronchodilator function, values of the FEV1 and FEV1/FVC% measurements in both COPD and CS groups (but not in the CNS group) prebronchodilator were repeated 20 min after the inhalation of 0.4 mg of salbutamol. The airflow obstruction was defined as reversible when the post-BD FEV1 values were increased compared to the pre-BD FEV1 values of at least 200 mL and 12%.

### 4.3. Collection and Processing of the Peripheral Lung Tissue

Twenty-eight subjects undergoing lung resection surgery for a solitary peripheral neoplasm were recruited. Lung tissue processing was performed as previously described [28,29]. Two to four randomly selected tissue blocks were taken from the subpleural parenchyma of the lobe obtained at surgery, avoiding areas grossly invaded by the tumor. Samples were fixed in 4% neutral formaldehyde in phosphate-buffered saline (PBS) at pH 7.2 and, after dehydration, embedded in paraffin wax. Serial sections 5 μm thick were first cut and stained with hematoxylin-eosin (H&E) in order to visualize the morphology and to exclude the presence of microscopically evident tumor infiltration. Tissue specimens were then cut for immunohistochemical analysis and placed on charged slides as previously reported [29].

### 4.4. Immunohistochemistry in Human Peripheral Lung Tissue

Immunostaining of paraffin-embedded peripheral lung tissue was performed as previously described [29].

For immunohistochemical analysis, 5-micron thick sections obtained from corresponding tissue blocks were deparaffinized, then washed in descending alcohol scale, treated with 3% hydrogen peroxide for 10 min, washed again in deionized water three times, and incubated with normal sheep serum to prevent unspecific adherence of serum proteins for 30 min at room temperature. Subsequently, sections were washed with deionized water and incubated for 30 min at 37 °C with commercially obtained primary anti-human antisera against different ATGs (see Table 3 and Table S1). Specifically, the primary antibody working dilution ratio able to affect the intensity of immunoreactivity has been determined after an accurate scalar dilution test in order to obtain the best specific results without any stained background. Next, the sections were washed three times with PBS and incubated in a Ventana BenchMark Ultra (Roche Diagnostics, Rotkreuz, Switzerland). An UltraView Universal DAB detection kit (Roche Diagnostics) was used in accordance with the manufacturer’s instructions. Slides were then removed from the Autostainer Ventana BenchMark Ultra (Roche Diagnostics, Rotkreuz, Switzerland), counterstained with Mayer’s Hematoxylin, mounted with Permount (Fisher Chemical, Rangeland Parkway, FL, USA), and coverslipped. Negative controls were obtained by omitting the specific primary antisera, replacing them with PBS or normal rabbit serum. In this way, no immunoreaction was revealed. By contrast, the positive control was represented by gastric cancer cells, since they were tested and stained by ATGs in a previous study with similar results to those elsewhere reported [30].

The immunoreactivity of ATGs was evaluated according to the intensity and percentage of positively stained cells, as elsewhere reported [1,31]. The cytoplasmic immunostaining intensity was rated as follows: 0, negative; 1, weak; and 2, strong. The percentage of positively stained cells was graded as follows: grade 0, 0–5%; grade 1, >5–25%; grade 2, >25–50%; grade 3, >50–75%; and grade 4, >75–100% for all antibodies. The immunohistochemical staining samples were independently scored by two pathologists (AI and GT), who were blinded to patient outcomes and other clinical findings, using a Zeiss Axioskop microscope (Carl Zeiss Microscopy GmbH, Jena, Germany) at 40× objective magnification. The interobserver agreement for immunohistochemistry staining had a kappa value ranging from 0.73–0.80 (substantial agreement) for the antisera.

The immunoreactive score was calculated by adding the staining intensity score and the percentage score of positively stained cells (0–6). Lung tissue compartments with an immunoreactive score of 0–3 were classified as negative, and those with a score of >3 were classified as positive. Autophagy was defined when samples were positive for at least two out of the three protein expressions [31].

### 4.5. Statistical Analysis

Group data were expressed as mean (standard deviation) for functional data or median (range) or interquartile range (IQR) for morphologic data. Differences between groups were analyzed using analysis of variance (ANOVA) for functional data. The ANOVA test was followed by the unpaired *t*-test for comparison between groups. The Kruskal–Wallis test applied for morphologic data was followed by the Mann–Whitney U test for comparison between groups. Probability values of *p* < 0.05 were considered significant. Data analysis was performed using the Stat View SE version 5.0, Graphics program (Abacus Concepts Inc., Berkeley, CA, USA).

## 5. Conclusions

It can be stressed that impaired activation of autophagy is mainly focused on both bronchiolar epithelium and the alveolar lining, which represent the most involved lung compartment in COPD. In light of these observations, we can hypothesize that COPD, ATGs, and ATG4 isoforms may be considered as additional potential targets for the development of new drugs, mainly addressed to chronic or degenerative lung disorders. Although we tested several ATG proteins, a major part of these did not change between COPD patients and controls; however, these results may enhance these findings as the selective increase of mainly ATG4 family proteins can explain the unbalance on the autophagic flux with an excessive ATG4 activity and thus an ineffective intracellular autophagy, whereas a global increase of all ATG proteins including also LC3B should be correlated to an increased autophagic flux. Therefore, on the basis of morphological and immunohistochemical results concerning the specific ATG4 family, strongly demonstrated in bronchial epithelium as well as alveolar lineage, the intriguing future direction of research may be addressed to develop some targeted specific therapeutic agents fighting with the autophagic flux in order to improve the clinic-prognostic course of COPD.

## Figures and Tables

**Table 1:** Immunohistochemical quantification of autophagic molecules in the peripheral lung of patients with COPD, in control smokers and non-smoking subjects.

Localization	Control Non-Smokers with Normal Lung Function	Control Smokers with Normal Lung Function	COPD Patients	Mann–Whitney U Test CNS vs. CS (*p* Value)	Mann–Whitney U Test CS vs. COPD (*p* Value)	Mann–Whitney U Test CNS vs. COPD (*p* Value)	Kruskal–Wallis (*p* Value)
Bronchiolar epithelium (score 0–6)							
ATG4A	1.6 (0–4)	0.90 (0–3)	3.5 (2–6) #*	0.4389	0.0006	0.038	0.0047
ATG4D	1 (0–3)	1.6 (0–6)	3.2 (1–6) #*	0.5249	0.0337	0.008	0.018
ATG5	1.2 (0–3)	1.1 (0–4)	3.1 (1–6) #*	0.8493	0.010	0.026	0.019
Alveolar septa (score 0–6)							
ATG4A	1.4 (0–3)	1.7 (0–6)	4.5 (2–6) #*	0.9424	0.010	0.0007	0.0036
ATG4B	1.9 (0–4)	1.1 (0–6)	4 (3–6) #*	0.1869	0.0061	0.0084	0.0064
ATG4C	1.1 (0–3)	1 (0–4)	3.5 (2–6) #*	>0.99	0.0032	0.016	0.0064
ATG4D	1.4 (0–3)	1.7 (0–6)	4 (2–6) #*	0.9763	0.0086	0.0053	0.0084
ATG5	1.4 (0–4)	1.1 (0–4)	3.7 (1–6) #*	0.7610	0.0050	0.015	0.0088
ATG7	2 (0–4)	1.5 (0–6)	4.2 (1–6) #*	0.4750	0.0111	0.0327	0.018

Scored (0–6) data expressed as mean value and range; Mann–Whitney U test: * significantly different from control non-smokers (CNS); # significantly different from control smokers (CS).

**Table 2:** Characteristics of subjects.

Group	N.	Age (Mean ± SD)	Male/Female	p-y	Ex/Current Smokers	Pre-BD-FEV1%	Post-BD-FEV1%	FEV1/FVC%
CNS	8	75 ± 9	3/5			113 ± 18%	ND	79 ± 5%
CS	10	66 ± 8	5/5	30 ± 13	8/2	100 ± 5%	ND	77 ± 8%
COPD	10	71 ± 3	9/1	50 ± 26	8/2	74 ± 16% *^#^	79 ± 16% *	62 ± 6% *^#^

Data are expressed as n or mean ± SD. For patients with COPD, forced expiratory volume in 1 s (FEV1)/forced vital capacity (FVC) are post-bronchodilator (β2) values. ND: not determined. *: significantly different from control smokers with normal lung function (ANOVA); #: significantly different from control never-smokers (ANOVA).

**Table 3:** Characteristics of primary antibodies utilized to identify autophagy signaling pathway components in the peripheral lung.

Primary Antibody	Manufacturer	Catalogue Number	Source/Host	Working Dilution	Positive Control
ATG2A	Atlas *	HPA038715	Rabbit	1:700	Gastric cancer cells
ATG2B	Atlas *	HPA001427	Rabbit	1:50	Gastric cancer cells
ATG3	Atlas *	HPA040471	Rabbit	1:300	Gastric cancer cells
ATG4A	Atlas *	HPA036374	Rabbit	1:300	Gastric cancer cells
ATG4B	Invitrogen **	PA5-30462	Rabbit	1:500	Gastric cancer cells
ATG4C	Atlas *	HPA007049	Rabbit	1:50	Gastric cancer cells
ATG4D	Atlas *	HPA067683	Rabbit	1:50	Gastric cancer cells
ATG5	Atlas *	HPA042973	Rabbit	1:100	Gastric cancer cells
ATG7	Atlas *	HPA007639	Rabbit	1:300	Gastric cancer cells
ATG10	Atlas *	HPA044163	Rabbit	1:100	Gastric cancer cells
ATG12	Invitrogen **	PA5-32180	Rabbit	1:500	Gastric cancer cells
ATG14 (Thr429)	Invitrogen **	PA5-105670	Rabbit	1:200	Gastric cancer cells
ATG14	Invitrogen **	PA5-78833	Mouse	1:500	Gastric cancer cells
ATG16L	Atlas *	HPA063900	Rabbit	1.300	Gastric cancer cells

Scoring system for immunohistochemistry in the peripheral lung tissue. * Solna/Stockolm, Sweden. ** Thermo Fisher Scientific, Waltham, MA, USA.

## Data Availability

All data generated or analyzed during this study are included in this article. Further enquiries can be directed to the corresponding author.

## Supplementary Materials

The following supporting information can be downloaded at: https://www.mdpi.com/article/10.3390/ijms25158182/s1.
