# Supplementary material for: Role of ATG4 Autophagy-Related Protein Family in the Lower Airways of Patients with Stable COPD
Source: Int J Mol Sci. 2024 Jul 26;25(15):8182. doi: 10.3390/ijms25158182 (PMC11311497; doi:10.3390/ijms25158182)
Supplement: Supplementary file 1 [file ijms-25-08182-s001.zip › ijms-3099021-supplementary.pdf]

Supplemental table S1. Immunohistochemical quantification of autophagic molecules that did not differ between the peripheral lung of patients with COPD, in control smokers and non-smoking subjects

Localization	Control non smokers with normal lung function	Control smokers with normal lung function	COPD patients	Kruskal Wallis (p value)
**Bronchiolar epithelium** (score 0-6)				
ATG2A	1.75 (0-4)	0.9 (0-4)	1.5 (0-4)	0.3160
ATG2B	1.5 (0-4)	1.5 (0-4)	1.5 (0-4)	0.9552
ATG3	1.6 (0-4)	1.2 (0-4)	1.7 (0-4)	0.5787
ATG4B	1 (0-4)	0.7 (0-4)	1.7 (0-4)	0.097
ATG4C	1 (0-4)	0.9 (0-4)	1.9 (0-4)	0.219
ATG7	1.75 (0-4)	1 (0-4)	1.8 (0-4)	0.4214
ATG10	1.25 (0-4)	0.8 (0-3)	1.9 (0-4)	0.2059
ATG12	1.6 (0-4)	0.7 (0-4)	1.5 (0-4)	0.3137
ATG14thr	1.3 (0-4)	0.8 (0-4)	1.8 (0-4)	0.2915
ATG14	0 (0-0)	0.1 (0-1)	0.2 (0-1)	0.4437
ATG16L	0.22 (0-1)	0.3 (0-2)	0.8 (0-2)	0.3726
**Alveolar septa** (score 0-6)				
ATG2A	1.8 (0-4)	1.1 (0-4)	1.8 (0-4)	0.4684
ATG2B	1.6 (0-4)	1.7 (0-4)	1.8 (0-4)	0.9407
ATG3	1.6 (0-4)	1.2 (0-4)	1.8 (0-4)	0.7716
ATG10	1.25 (0-4)	0.8 (0-3)	1.9 (0-4)	0.3969
ATG12	1.6 (0-4)	0.8 (0-4)	1.6 (0-4)	0.3460
ATG14thr	1.3 (0-4)	0.8 (0-4)	1.9 (0-4)	0.2916
ATG14	0.3 (0-1)	0.1 (0-1)	0.9 (0-2)	0.093
ATG16L	0.12 (0-1)	0.3 (0-2)	0.8 (0-2)	0.2622

Scored (0-6) data expressed as mean value and range; Mann Whitney U test: * significantly different from control non smokers (CNS); # significantly different from control smokers (CS); NE=not evaluable.
